# Sequential Improvement from Cosolvents Ink Formulation to Vacuum Annealing for Ink-Jet Printed Quantum-Dot Light-Emitting Diodes

**DOI:** 10.3390/ma13214754

**Published:** 2020-10-24

**Authors:** Young Joon Han, Do Yeob Kim, Kunsik An, Kyung-Tae Kang, Byeong-Kwon Ju, Kwan Hyun Cho

**Affiliations:** 1 Manufacturing Process Platform Research and Development Department, Korea Institute of Industrial Technology (KITECH), 143 Hanggaul-ro, Sangnok-gu, Ansan-si 15588, Korea; youngjhan@kitech.re.kr (Y.J.H.); ehduq077@kitech.re.kr (D.Y.K.); kunsik1214@kitech.re.kr (K.A.); ktkang@kitech.re.kr (K.-T.K.); 2 Department of Electrical and Electronics Engineering, College of Engineering, Korea University, 145 Anam-ro, Seongbuk-gu, Seoul 02841, Korea

**Keywords:** quantum-dot (QD), quantum-dot light-emitting diodes (QLEDs), ink-jet printing, cosolvents, vacuum annealing

## Abstract

Optimization of ink-jet printing conditions of quantum-dot (QD) ink by cosolvent process and improvement of quantum-dot light-emitting diodes (QLEDs) characteristics assisted by vacuum annealing were analyzed in this research. A cosolvent process of hexane and ortho-dichlorobenzene (oDCB) was optimized at the ratio of 1:2, and ink-jetting properties were analyzed using the Ohnesorge number based on the parameters of viscosity and surface tension. However, we found that these cosolvents systems cause an increase in the boiling point and a decrease in the vapor pressure, which influence the annealing characteristics of the QD emission layer (EML). Therefore, we investigated QLEDs’ performance depending on the annealing condition for ink-jet printed QD EML prepared using cosolvents systems of hexane and oDCB. We enhanced the quality of QD EML and device performance of QLEDs by a vacuum annealing process, which was used to prevent exposure to moisture and oxygen and to promote effective evaporation of solvent in QD EML. As a result, the characteristics of QLEDs formed using ink-jet printed QD EML annealed under vacuum environment increased luminescence (L), current efficiency (CE), external quantum efficiency (EQE), and lifetime (LT_50_) by 30.51%, 33.7%, 21.70%, and 181.97%, respectively, compared to QLEDs annealed under air environment.

## 1. Introduction

Recently, quantum-dot light-emitting diodes (QLEDs) using the electroluminescence (EL) property of quantum-dot (QD) have been actively studied as next generation alternative light-emitting devices (LEDs) [1,2,3,4]. The high color purity and good stability of QDs make them very suitable for use in LEDs. Above all, the ability to obtain a desired wavelength of light by adjusting the core diameter of the QDs is the driving force of many QLED studies. However, device performance factors such as efficiency and lifetime of QLEDs have not reached the level of commercialized organic light-emitting diodes (OLEDs). Unlike organic layer vapor deposition in OLEDs, QDs have a limitation in that a film must be formed by a solution process such as spin-coating, transfer printing [5,6,7], or ink-jet printing [8,9,10]. Among various solution processes, the ink-jet printing process has been attracting much attention as a method of effectively reducing the material cost, shortening the process times, and forming desired films regardless of substrate size and type [11,12,13,14]. In recent years, research on forming QD emission layers (EMLs) by ink-jet printing has been actively performed. In order to form a QD EML by ink-jet printing, it is necessary to understand the properties of inks used in conventional ink-jetting. Control of solution properties of the inks is required for a stable ink-jetting process; these properties include viscosity, surface tension, and density of the solution. There are many factors to be considered for stable jetting of a droplet of ink, such as the diameter of the cartridge nozzle to be used for ink-jetting, the jetting speed of droplets, and the distance between the cartridge nozzle and the substrate. However, in the conventional ink-jetting process, without additives, viscosity, and surface tension properties of common organic solvents used to disperse QDs are not suitable for obtaining ink with properties making it capable of ink-jetting. In order to solve this problem, the cosolvent process proposed in this study combines, at a specific ratio, organic solvents having two different solution properties, leading to an effective method of controlling the solution properties of ink. For the formation of good properties of film in OLED or QLED fabrication by ink-jet printing with the cosolvent process, Z. Ding et al. have reported on OLEDs that improve the properties of ink-jet films printed by cosolvent process [15]; C. Jiang et al. have reported on QLEDs that minimize the coffee-ring effect of droplets by cosolvent process [16]. In this study, utilizing the cosolvent process to control the solution properties of QD ink, QD ink droplets have been optimized to a constant, size and volume, without tail and satellite droplets, for stable ink-jetting.

**Figure 1:**
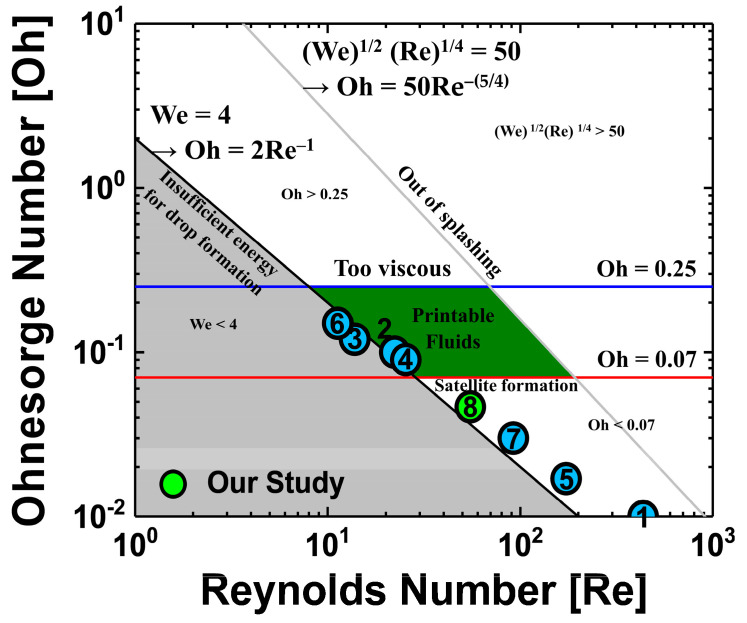
Ohnesorge map of the cosolvent QD inks used in previously studies and our study. For printable fluids region, which are green areas in the center of the graph, the ink is determined to have stable ink-jetting properties if there is dot in this area. Each dot with numbers on the Ohnesorge map represents the cosolvents QD inks from Table 1.

Figure 1 provides an Ohnesorge map, made by calculating the Reynolds number, Weber number, and Ohnesorge number and taking into account the parameters of the cosolvent QD inks used in previously reported studies [8,9,10,16,17,18,19] and our study. Each dimensionless number of these references is listed in Table 1. The aim of the studies mentioned in Figure 1 is the optimization of ink-jet printing by cosolvent method of two different solvents; various solvents were used, and the mix ratio of used solvents and the annealing conditions for residual solvent evaporation were also different. Interestingly, Figure 1 shows that not all of the studies have dimensionless numbers in the printable fluid region. However, dimensionless numbers should not be ignored because they are very helpful in designing ink formations by referencing the viscosity and surface tension of the ink with the other jetting conditions (cartridge, applied voltage waveform, etc.). Not only the solution properties of the ink, but also other factors, such as the temperature of the cartridge nozzle, the distance from the nozzle to the substrate, and the applied voltage waveform were used to optimize the process. Therefore, experiments to consider cosolvent process conditions of QD ink were designed such that the cosolvents QDs ink properties were as close as possible to those designated in the printable fluid region on the Ohnesorge map. Two solvents used in the cosolvent process usually have different dewetting velocities, resulting in opposite characteristics of viscosity, surface tension, boiling point, and vapor pressure. In the ink selection process for ink-jet printing, a parameter that should be considered with special care is the boiling point, because a high boiling point of the solvent when drying the ink-jet printed film may cause deterioration of the film properties by the residual solvent. In the previously reported studies, detailed analysis about the annealing process using cosolvent inks on QLEDs performance was insufficient. Because they focused more on making stable ink-jetting and fabricating the uniform film without coffee-ring using cosolvent inks, QLEDs’ performances such as current efficiency (CE) and external quantum efficiency (EQE) have not been studied in detail as shown in Table 1. In addition, the result of a lifetime measurement of the QLED fabricated by ink-jet printing using cosolvents ink has not been reported. In this work, the vacuum annealing process was optimized for effective evaporation of residual solvent of the ink-jet printed film, and the QLEDs performances were observed by measuring the lifetime depending on the annealing environment of QD EML.

In this study, the formation of QD EML pixels was optimized in successive processes of ink-jet printing, from ink formulation to annealing conditions. To achieve stable ink-jetting and uniform film properties, we studied the control of solution properties of QD ink by cosolvent process based on conventional ink-jetting logic theory. The ink-jetting conditions of the QD ink were optimized by a cosolvent process in which the mixing ratio of hexane and ortho-dichlorobenzene (oDCB) was adjusted to match the specifications of the cartridge used in this experiment. In addition, it was observed that problems of surface roughness and dewetting on the surface of QD EML pixels, and pile-up at walls of the ink-jet printed QD EML pixels, were solved by the cosolvent process. Next, to observe the effect of vacuum annealing on the ink-jet printed QD EML, the electrical, optical, and lifetime characteristics of ink-jet printed QLEDs annealed under air, dinitrogen (N_2_), and vacuum condition were compared.

## 2. Materials and Methods

### 2.1. Materials

For hole injection layer (HIL) material, poly (3,4–ethylenedioxythiophene):poly (styrene sulfonate) (PEDOT:PSS) (Clevios P VP AI 4083, Heraeus Co., Hanau, Germany) was mixed with isopropyl alcohol (IPA) in a ratio of 1:1, and sonication was performed at room temperature (RT) for 5 min. Next, poly (9,9–dioctylfluorene–co–*N*–(4–(3–methylpropyl)) diphenylamine) (TFB) (OSM Co., Goyang, Korea) was selected as hole transport layer (HTL) was dispersed in toluene (Samchun Chemical Co., Pyeongtaek, Korea) at a concentration of 8 mg/mL, and then stirred for 24 h at 50 °C and 400 rpm. For EML material, QDs of CdZnSeS core/ZnS shell structure were purchased from In-visible Co. (Suwon, Korea), and the ligand of the QDs was formed by mixing trioctylphosphine and oleic acid. The QDs were dispersed in solvents with four mix ratio conditions of (1) only hexane; hexane and oDCB at ratios of (2) 1:1 and (3) 1:2; and (4) only oDCB at a concentration of 20 mg/mL. The QD solution was stirred at 400 rpm for 2 h at RT and then refrigerated. Hexane and oDCB were purchased from Sigma-Aldrich (St. Louis, Missouri (USA), Germany).

### 2.2. Fabrication of QLEDs

First, PEDOT:PSS/IPA mixed solution used as HIL was spin-coated at 2000 rpm for 30 s on the cleaned indium-tin-oxide (ITO) coated glass substrate, and then annealed at 100 °C for 30 min. Next, TFB solution of concentration of 8mg/mL was spin-coated at 4000 rpm for 30 s and annealed at 150 °C for 30 min. TFB film was used as HTL. After that, in order to compare the performance of QLEDs depending on process method, the QD EML was formed by two solution methods, either spin-coating or ink-jet printing. In the case of the spin-coating process, the QD solution was spin-coated at 5000 rpm for 30 s and annealed at 115 °C for 30 min. In the case of the ink-jet printing process, QD ink was ink-jet printed using a DIMATIX DMP 2800 (Fujifilm Co., Tokyo, Japan) ink-jet printer with DMC-11610 cartridge (Fujifilm Co., Tokyo, Japan) under air atmosphere; cartridge nozzles and substrates were maintained in an un-heated state. The distance from the nozzle to the substrate was 0.6 mm, the speed of the droplet was about 3 m/s, and the time per cycle of the applied voltage waveform was about 50 μs. Through fine adjustment with proper nozzle purging, the applied voltage was selected from a range between 10 and 10.5 V. After ink-jet printing, QD EML was annealed at 115 °C for 30 min under air, N_2_, and vacuum atmosphere. The entire solution process was carried out in air at RT. Next, 1, 3, 5–tris (2–*N*–phenylbenzimidazolyl) benzene (TPBi), lithium fluoride (LiF), and aluminum (Al) used as the electron transport layer (ETL) and cathode electrode, respectively, were sequentially deposited by thermal evaporation. TPBi was deposited to a thickness of 25 nm at 0.8 Å/s, and LiF and Al were deposited to a thickness of 1 nm and 100 nm at 0.5 Å/s and 2 Å/s, respectively. The thermal evaporation process was conducted under high vacuum pressure of 2 × 10^−7^ Torr. Finally, after the thermal evaporation process, all fabricated devices were encapsulated in N_2_ atmospheric conditions.

### 2.3. Characterization and Measurements

The ink-jet printed QLEDs were encapsulated by ejecting a XNR5570-B1 resin (Nagase ChemteX Co., Osaka, Japan) using an S-SIGMA-CM3-V5 dispenser (Musasi Engineering Inc., Tokyo, Japan) with a SHOTMASTER300ΩX tabletop robot (Musasi Engineering Inc., Tokyo, Japan). The viscosity levels of the QD inks prepared under each cosolvent process condition were measured at a shear rate of 8000 s^−1^ at RT with a μVISC Portable Viscometer (RheoSense Inc., San Ramon, CA, USA). An Alpha-Step IQ (KLA-Tencor Co., Milpitas, CA, USA) was used to measure the height of the QD EML pixels. An NX10 (Park Systems Co., Seongnam, Korea) was used to measure the surface roughness of the QD EML pixel. An M6100 OLED I-V-L Test System (McScience Co., Suwon, Korea) and an M6000 plus OLED Lifetime Test System (McScience Co., Suwon, Korea) were used to measure the current-voltage-luminance (IVL) characteristics and lifetime of QLEDs, respectively. C11347-11 (Modoo Technology Co., Seoul, Korea) was used to measure the photo-luminescence-quantum yield (PLQY) characteristics of the ink-jet printed QD EML.

## 3. Results and Discussion

In a solution process using ink-jet printing, the following three dimensionless numbers are used to design the properties of inks suitable for ink-jetting [20,21]. These dimensionless numbers are expressed as:

(1) Reynolds number (Re) = υρaη

(2) $$\mathrm{Weber}\;\mathrm{number}\;(\mathrm{We})\;=\;\frac{\upsilon ²\rho a}{\gamma }$$

(3) Ohnesorge number (Oh) = WeRe = ηγρa

The value *υ* is the speed of a droplet, *ρ* is the density of the fluid, *a* is the diameter of the nozzle, *γ* is the surface tension of the ink, and *η* is the viscosity of the ink. The Reynolds number is the ratio of force due to inertia to force due to viscosity and quantitatively indicates the relative importance of these two forces in a given flow condition. The Weber number can be considered a measure of the relative importance of fluid inertia compared to surface tension. This value is useful for analyzing the formation of droplets and bubbles. The Reynolds number and Weber number are two of the most important dimensionless numbers in fluid dynamics and are used with other dimensionless numbers to determine the dynamic similitude. The viscosity and surface tension of the ink in the ink-jetting process are very important in the process of forming and jetting droplets stably from nozzles. The ratio of Reynolds number to Weber number is the Ohnesorge number. The speed term of a droplet ejected from the nozzle term is erased so that the number is composed of the ratio of the viscosity of the ink to the surface tension. As introduced earlier, the Weber number is a dimensionless number related to the surface tension, and the Reynolds number, related to the viscosity, is set to an appropriate range for ejecting ink from the nozzle. If the Weber number is less than 4, the droplet is insufficient for jetting from the nozzle. If (We)^1/2^(Re)^1/4^ is larger than 50, the ink droplet ejected from the nozzle will splash without maintaining a sphere shape. Even if the Weber number is a dimensionless number related to the surface tension, it is also affected by other factors (speed of droplet, density of fluid, diameter of nozzle) and should be considered. When only the surface tension of the ink is adjusted, it may be difficult to obtain stable ink-jetting properties and to form suitable droplets. Therefore, the Ohnesorge number must also be used in the optimization of inks. Previous studies have shown that when the Ohnesorge number is less than 0.1 or 0.07, satellite droplets are generated; when the Ohnesorge number is greater than 0.25 or 1, droplet tails are lengthened due to excessive viscosity [20]. In this experiment, Ohnesorge numbers between 0.07 and 0.25 were chosen as the optimal range for stable jetting.

**Figure 2:**
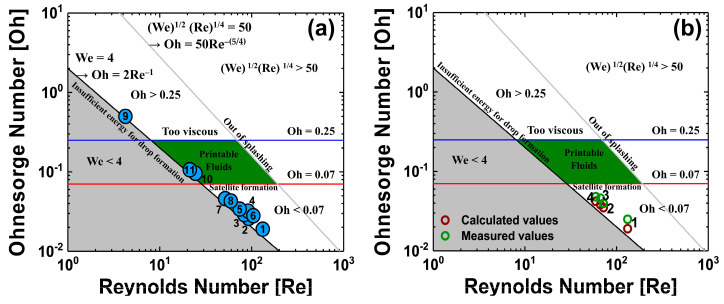
Ohnesorge map of (**a**) each organic solvents and (**b**) cosolvents inks of each mix ratio. Each dot with numbers on the Ohnesorge map represents the organic solvents from Table 2 and Table 3, respectively.

Figure 2a shows the Ohnesorge map, obtained through calculation of the dimensionless numbers and by considering the parameters of organic solvents commonly used in solution processes. The parameters of the organic solvents are listed in Table 2. Figure 2a shows that cyclohexylbenzene (CHB) and a-chloronaphthalene (ClNaph) are in the jettable range for ink-jetting with a single solvent, without the cosolvent process. However, the solvents used for ink-jet printing of QD EML should have low boiling point properties to avoid thermal degradation of QDs under the annealing process for solvent evaporation [22,23]. This is because excessively high temperatures can lead to QD surface defects due to non-radiative recombination. CHB and ClNaph have high boiling points and were not considered in this experiment. Hexane, used in our previous studies [24], has a low boiling point, which is advantageous for achieving effective solvent evaporation with annealing of QD EML. However, the low viscosity of hexane makes it unsuitable for use as a single solvent in ink-jet printing, and so the cosolvent process with oDCB was used to control the ink solution properties. In the QD EML annealing process for solvent evaporation, the high boiling point of oDCB can be disadvantageous for complete evaporation of the solvent; however, this property of oDCB could compensate for the poor viscosity and surface tension of hexane. The solution properties, which depend on the mix ratio of hexane and oDCB, are shown in Table 3. The properties of each mixed solvent’s condition, considering the calculated dimensionless numbers are shown in Figure 2b. In the case of viscosity, which is the dominant characteristic in QD ink production, the dimensionless numbers obtained using the viscosity values in the literature and those obtained using the viscosity values measured in the experiment were compared. It was found that the actual measured viscosity value was higher than the viscosity values in the literature; the measured value can be seen to be within the error range according to the measurement conditions. Considering only dimensionless numbers, using oDCB as a single solvent may be a good choice. However, considering surface tension values suitable for the cartridges used in this experiment, it was expected that the best ink properties would be obtained when hexane and oDCB were mixed at a ratio of 1:2. Unfortunately, the dimensionless numbers of the four cosolvents condition considered in this experiment do not meet the jettable range on the Ohnesorge map.

**Figure 3:**
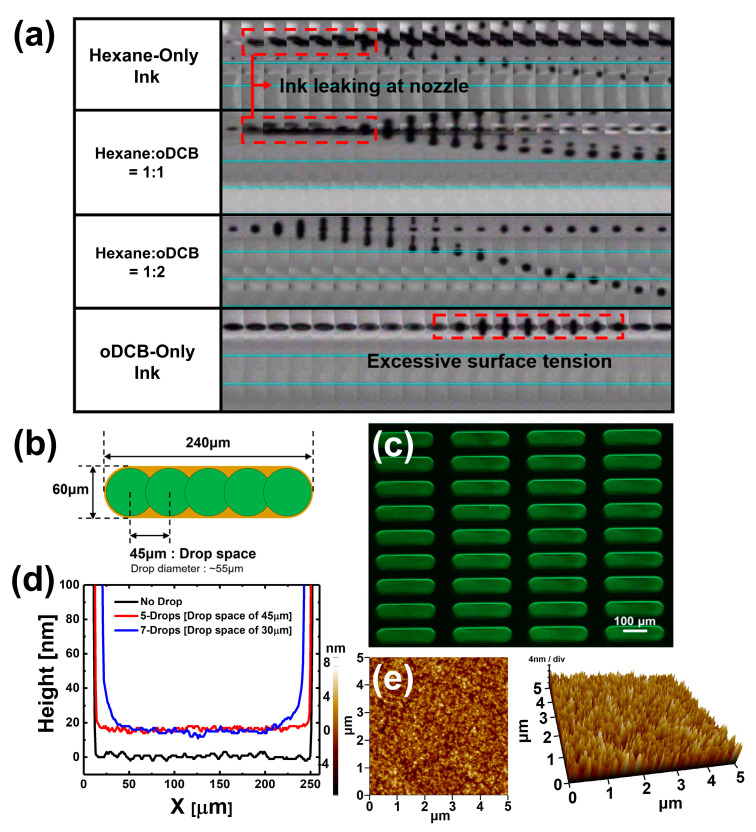
(**a**) Ink-jetting motion capture for each cosolvent condition. (**b**) Bank pixel information and 5-drop space conditions. (**c**) OM images with UV light of QD EML pixels. (**d**) Height profile of the QD EML by ink-jet printing of cosolvents QDs ink of hexane and oDCB with ratio of 1:2 under 5-drop and 7-drop space conditions. (**e**) AFM images of the QD EML pixel surface. Surface roughness RMS value was measured 1.59 nm.

However, the results in Figure 3 show that a QD EML can be formed in a pixel with cosolvent inks using only organic solvents without any other additives. Figure 3a shows ink-jetting motion depending on the four different cosolvent conditions. In the case of the hexane-only ink, the ink leaked around the nozzle and the jetting direction of ejected droplets was twisted. This phenomenon appears to be due to non-uniformity of the viscosity and surface tension of the nozzle edging caused by ink leaking around the nozzle. Therefore, the jetting direction of the droplet ejected from the nozzle twisted randomly; this type of jetting condition is not reliable in terms of accuracy of position of droplet reaching the substrate. For the jetting condition using cosolvent QD inks, in which hexane and oDCB were mixed at a ratio of 1:1, ink also leaked around the nozzles, similar to the jetting condition when using the hexane-only ink. Although the jetting direction of the ejected droplet was not twisted, due to insufficient viscosity of the cosolvents QDs ink, satellite droplets were unable to join the main droplet and jetted separately. Since such satellite droplets drop outside the desired area, they are not suitable for achieving the original purpose of ink-jet printing and may adversely affect the jetting direction of the ejected droplet, twisting it. On the other hand, when hexane and oDCB were mixed at a ratio of 1:2, the cosolvent QD ink did not leak around the nozzle and maintained a constant jetting direction of ejected droplet. Although there was a problem that satellite droplets remained at the tail of the main droplet, the satellite droplets joined the main droplet and constant ink-jetting was maintained. Finally, in the case of ink-jetting condition using oDCB-only ink, excessive surface tension of the ink obstructed droplet ejection from the nozzle and, when the waveform voltage was increased, the droplet was sprayed by excessive ejection pressure. The QD EML pixel information and the 5-drop process condition are shown in Figure 3b. The active pixel has an area of 60 μm × 240 μm (80 pixels per inch, ppi) and fills one pixel with 5-drop ink-jet printing in a 45 μm drop space using cosolvent QD ink of hexane and oDCB at a ratio of 1:2.

Figure 3c is a photograph taken with an optical microscope (OM) with ultraviolet (UV) light after forming QD EML pixels by ink-jet printing. It can be seen that the QD ink fills the pixels well, without messy surface caused by satellite droplets. In addition, it was confirmed that dewetting and pinholes did not occur on the QD EML pixel surface. For this reason, if the boiling point of the solvent is high, it helps to form a film of higher thickness, which can suppress dewetting of the ink-jet printed film from a thermodynamic aspect. In addition, if the viscosity of the solvent is high, dewetting of the ink-jet printed film can be suppressed from a kinetic aspect. There have been reports of good quality film formation achieved using the different viscosities and boiling points of the two different solvents [15]. In this experiment, this interaction helped to form QD EML without dewetting or pinholes. Figure 3d shows the height profile of ink-jet printed QD EML; the height of the two ink-jet printed pixels can be compared under the two conditions of 5-drops and 7-drops. In order to use ink-jet printing to print fine patterns, it is most important to control the surface energy of the substrate to optimize the spread of the droplets; however, it is also necessary to control the spacing between droplets, so that the droplets overlap properly with each other. It is also important to prevent bulging, in which later-printed droplets affect the position of previously printed droplets, so that patterns cannot be aligned in line [25]. In this experiment, the film properties of QD EML pixels were observed under ink-jet printing conditions of drop spaces of 5-drops and 7-drops. The heights of both drop space conditions of the QD EML pixel were measured at about 20 nm; however, for the 7-drop condition, the ink piled up on the polymer partition wall. According to the inherent properties of the particles in a liquid, when a liquid is present in an interior space surrounded by a solid interface, the phenomenon of flow of particles in the liquid through the solid interface wall surrounding the interior is called the pile-up effect [26]. As can be seen in Figure 3e, the surface roughness root-mean-square (RMS) value of the QD EML pixel surface was measured by atomic forced measurement (AFM) and found to be 1.59 nm; the formation of QD EML pixels by ink-jet printing was successful because the surface roughness RMS value was not a hurdle to the fabrication of multi-layered QLEDs.

**Figure 4:**
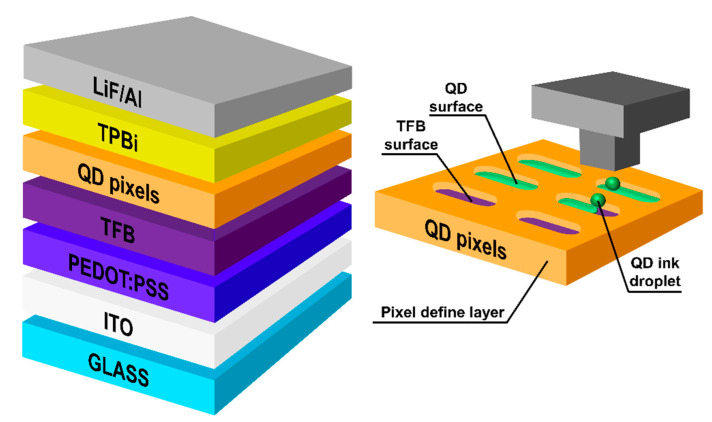
Schematics of ink-jet printed QLED structure and ink-jet printed QD EML pixel arrays.

Figure 4 provides a schematic of the ink-jet printed QLEDs. Toluene, used to disperse the TFB film used as HTL, is not an orthogonal solvent with hexane and oDCB in QD EML. The organic solvents used in the formation of the two layers (HTL and QD EML) may interact with each other and adversely affect QLEDs, by such processes as dissolving, intermixing, pinhole formation, and dewetting; however, in our previous research, such processes were not found in TFB under 150 °C annealing conditions [24]. In this experiment, the annealing temperature of TFB was fixed at 150 °C.

**Figure 5:**
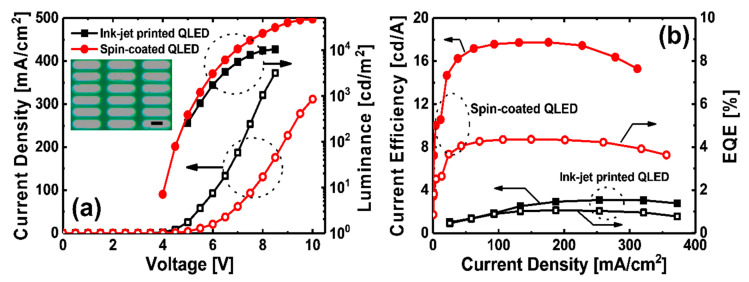
(**a**) Comparison of current density and luminance-voltage characteristics of spin-coated QLEDs and ink-jet printed QLEDs. (Inset: EL images of ink-jet printed QLEDs under applied voltage of 5 V. Scale bar: 100 μm) (**b**) Comparison of current efficiency and EQE-current density characteristics of spin-coated QLEDs and ink-jet printed QLEDs.

As confirmed by the OM image obtained with UV light, none of the pixels of the ink-jet printed QLEDs showed dissolving or pinhole formation, as can be seen in the electroluminescence (EL) image provided in inset of Figure 5a. This result shows that the sufficient hardness of the TFB film and the use of the cosolvent process of QD EML contributed to the prevention of adverse effects on film properties. However, the IVL characteristics comparison graph results shown in Figure 5a,b indicate that ink-jet printed QLEDs have lower IVL and efficiency characteristics than those of spin-coated QLEDs; this is to be expected though because the spin-coating process and ink-jet printing process have different solvent evaporation mechanisms during film formation. In the case of the spin-coating process, dynamic evaporation of the solvent by centrifugal force starts from the moment spinning begins, and most of the solvent evaporates during the first 30 s of spinning. On the other hand, in the case of the ink-jet printing process, static evaporation of the solvent starts from the moment a droplet is ink-jet printed and forms a film on the substrate; unlike spin-coating, there is no assistance by external forces in the solvent evaporation process. Therefore, although the annealing conditions are the same, differences in amount of solvents before the annealing process cause differences in quality of QD EML. A phase diagram of the vacuum annealing process, used to lower the boiling point of the solvent, is shown in Figure S1. Lowering the pressure around the solvent lowers the temperature at which the solvent evaporates. Since the vacuum atmospheric pressure has a lower torr value than that of normal atmospheric pressure, the boiling point of the solvent is also lowered; solvent in the QD EML can thus be minimized by vacuum annealing.

It is necessary to not only study the uniform and good properties of active pixels obtained by stable ink-jet printing process, but also improvement of lifetime, to allow devices to emit light stably and uniformly for a long time. W. Cao et al. have improved the lifetime of QLEDs by using QD EML with tailored energy band structures to improve the hole transport property between the highest occupied molecular orbital (HOMO) level of the HTL and the HOMO level of the QD EML [27]. Chang et al. have reported that degradation of QLEDs performance is due to two phenomena caused by a discrepancy of the hole-electron injection rate into the QDs [28]. First, the luminance efficiency of the QD EML is reduced due to accumulation of excessive electrons in QDs; the second is electron leakage, which causes non-radiative recombination centers to generate irreversible physical damage in the HTL [28]. Solvents in the QD EML may also cause problems in IVL characteristics and lifetime of LEDs [29,30,31,32]. To improve the characteristics of ink-jet printed QLEDs by effectively evaporating the solvents that cause degradation of film properties of QD EML, IVL and lifetime characteristics of QLEDs depending on annealing conditions of QD EML were analyzed.

**Figure 6:**
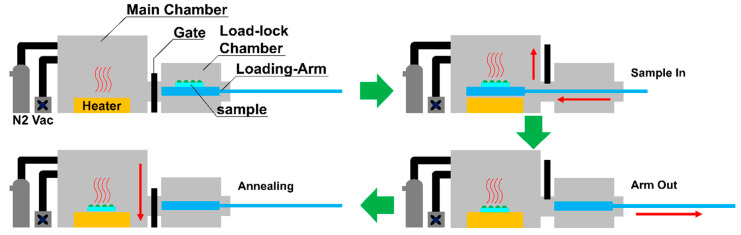
Schematic diagram of an annealing chamber for the annealing atmospheric conditions experiments of the QD EML pixel.

Figure 6 shows a simple process diagram of an annealing process used to vary the annealing conditions of QD EML. The equipment consists of a loading chamber for loading device samples and a main chamber for the annealing process. The QD EML were annealed under three different conditions: air, N_2_, and vacuum; each annealing process condition was precisely controlled to ensure that the process time, from sample loading to sample collection after annealing, was exactly the same. In addition, for a fair comparison of device characteristics depending on each annealing atmospheric condition of the QD EML, all other process conditions, except the annealing process of the QD EML (anode (ITO coated on glass substrate), HIL, HTL, ETL, and cathode) of QLEDs were kept identical. The annealing process using N_2_ removes moisture and oxygen in the air atmosphere and performs annealing in a closed chamber in which only the inert gas N_2_ circulates to prevent adverse effects of moisture and oxygen on the QD EML surface. Annealing in vacuum conditions can effectively evaporate solvents inside the QD EML, as demonstrated in previous studies [33], and can prevent degradation of film quality by external atmospheric components such as oxygen and moisture [32]. By controlling the annealing atmospheric condition of the QD EML to improve film quality, recombination in the QD EML of electron-hole pairs can be improved. In addition, the IVL characteristics and the lifetime degradation of QLEDs caused by electron accumulation effect between QD EML and ETL will be improved.

**Figure 7:**
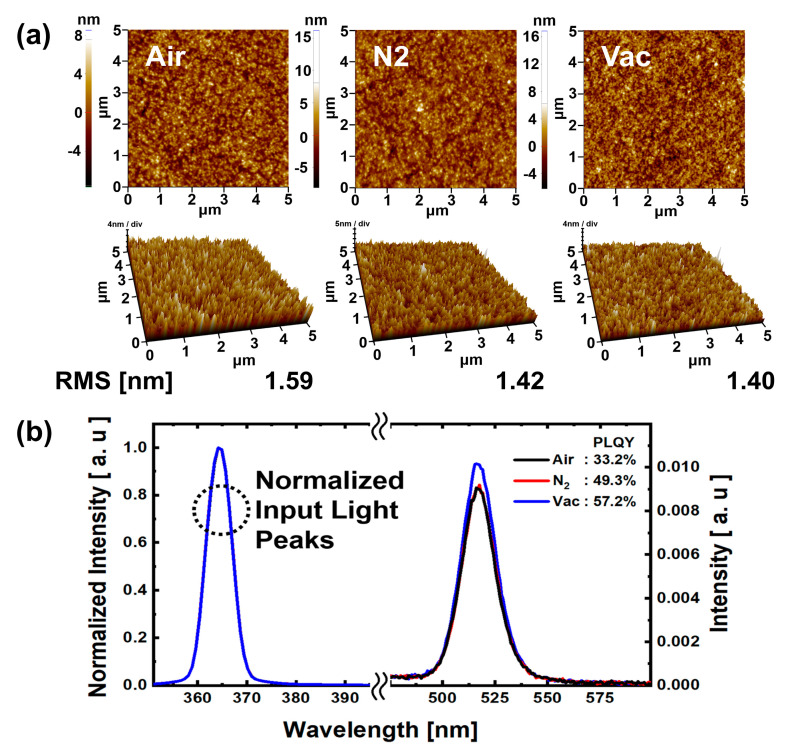
(**a**) AFM images and (**b**) PLQY characteristics of the QD EML pixel surface annealed under air, N_2_, and vacuum atmospheric conditions.

The AFM results in Figure 7a show that the surface roughness RMS values for the N_2_ and vacuum annealing conditions are smaller than those for the air annealing condition. This might be because annealing processes under N_2_ and vacuum conditions are less affected by external components than is the case for the air condition. Thus, it can be assumed that better QD EML film properties were obtained in the N_2_ and vacuum annealing conditions than in the air annealing condition. In Figure 7b, results of PLQY analysis are shown for QD EML under each annealing atmospheric condition. The QD EML surface was ink-jet printed with 45 μm drop space; the annealing process was the same as that used for the QD EML ink-jet printed QLEDs. It was confirmed that the photoluminescence peaks for all atmospheric condition annealed QD EML showed normal green emission at 525 nm. The PLQY values were 33.2%, 49.3%, and 57.2%, respectively, for air, N_2_, and vacuum annealing conditions. The PLQY values for the N_2_ and vacuum annealing conditions were 48.49% and 72.29% higher than those for the air annealing condition. As with the AFM analysis, low moisture and oxygen reduction in N_2_ and vacuum annealing conditions can prevent degradation of QDs [34,35], which may result in improved PLQY values. In particular, efficient solvent evaporation due to the high vacuum pressure of the vacuum annealing condition, compared to air and N_2_ annealing conditions, may also have contributed to the increase in the PLQY value.

**Figure 8:**
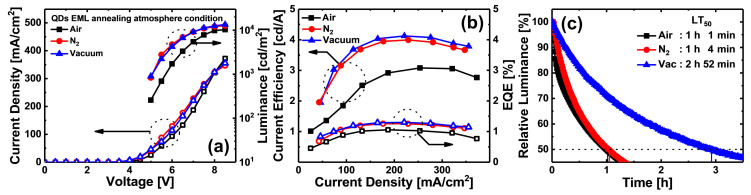
Comparison of (**a**) current density and luminance-voltage, (**b**) current efficiency and EQE-current density, and (**c**) LT_50_ characteristics of the ink-jet printed QLEDs annealed under each annealing atmospheric condition.

Figure 8 compares the IVL and lifetime characteristics of ink-jet printed QLEDs; the IVL and lifetime (LT_50_) characteristics showed different trends. Values of luminance, CE, EQE, and LT_50_ of QLEDs annealed under N_2_ condition increased by about 23.46% (12,719 cd/m^2^), 29.22% (3.98 cd/A), 19.81% (1.27%), and 4.9% (1 h 4 min), respectively, compared with QLEDs annealed under air condition. In the case of the QLEDs annealed under vacuum condition, values of luminance, CE, EQE, and LT_50_ increased by 30.51% (13,445 cd/m^2^), 33.77% (4.21 cd/A), 21.70% (1.29%), and 181.97% (2 h 52 min), respectively. In order to more accurately observe the decrease in normalized luminance over time, the LT_50_ of QLEDs is shorter because the constant voltage (CV) mode is used instead of the constant current (CC) mode, which is typically used for LED lifetime measurement. However, there was no problem in identifying trends for each annealing atmospheric condition, and when the QD EML of QLEDs was annealed in vacuum condition, the longest device lifetime was clearly identified. As with previous experimental results, the IVL and LT_50_ characteristics of QLEDs were the lowest in air annealing condition. The IVL characteristics of QLEDs annealed under N_2_ and vacuum conditions did not differ significantly from each other. However, comparing the LT_50_ values of QLEDs annealed under each atmospheric condition, the LT_50_ of the QLEDs annealed under N_2_ condition was not significantly different from the case of air annealing condition, but the QLEDs annealed under vacuum condition had an LT_50_ value about three times larger than those of the cases of air and N_2_ annealing conditions. The N_2_ annealing condition shows less degradation of QD EML because the QDs are less exposed to oxygen and moisture than they are in the air annealing condition. However, since there was no significant difference in atmospheric pressure between the air (740 mTorr) and N_2_ (780 mTorr) atmospheric conditions, the evaporation rates of the solvents were similar, such that the LT_50_ of QLEDs annealed under N_2_ condition did not improve compared to that of the QLEDs annealed under air condition. On the other hand, for the QLEDs annealed under vacuum condition, the QDs were not exposed to oxygen or moisture, and efficient solvent evaporation was probably achieved due to low atmospheric pressure in the annealing process.

## 4. Conclusions

In conclusion, the cosolvent process was used to optimize the ink-jetting conditions of QD ink, without any additives that could degrade the properties of the QD EML. Although the dimensionless number properties of the cosolvents QD ink of hexane and oDCB at a ratio of 1:2 did not enter the printable fluid region on the Ohnesorge map, it has been confirmed that the QD ink had suitable viscosity and surface tension characteristics for cartridge use because satellite droplets were minimized. Under optimized ink-jetting conditions, ink-jet printed QD EML pixels achieved uniform surface properties without dewetting or dissolving. Furthermore, the drop space between each droplet (45 μm) was controlled to prevent pile-up effects on the bank walls. To improve the performance of ink-jet printed QLEDs, the QD EML were annealed under air, N_2_, and vacuum conditions, and the IVL characteristics and LT_50_ values of the QLEDs under each annealing atmospheric condition were analyzed. As a result, by eliminating moisture and oxygen during annealing, both N_2_ annealing and vacuum annealing were found to improve the IVL characteristics of QLEDs. In particular, for vacuum annealed QLEDs, the LT_50_ increased by 181.97% because the low atmospheric pressure in the annealing environment caused solvent evaporation of QD EML; this result is expected to help solve the lifetime issue of QLEDs.

## Figures and Tables

**Table 1:** List of the solution properties of cosolvents’ QD inks and the device characteristics in previously reported researches (EG: ethylene glycol, L: luminance, CE: current efficiency, EQE: external quantum efficiency).

No.	Used Solvents and Mix Ratio	Re	We	Oh	Annealing Condition	L (cd/m^2^)	CE (cd/A)	EQE (%)	Lifetime (min)
1. [8]	Hexane:Octane = 10:1	434.73	22.91	0.01	50 °C (Not Mentioned)	-	-	-	-
2. [9]	CHB:Decane = 9:1	22.26	5.17	0.1	70 °C (Not Mentioned)	12,100	4.44	-	-
3. [10]	CHB:Octane = 95:5	13.81	2.71	0.12	70 °C (Not Mentioned)	3,050	2.8	2.4	-
4. [16]	CHB:oDCB = 8:2	25.4	5.41	0.09	Unheated, (Vacuum)	12,000	4.5	-	-
5. [17]	Water:EG = 9.8:0.2	173.12	8.83	0.017	70 °C (Not Mentioned)	-	-	-	-
6. [18]	Water:EG = 7:3	11.29	2.88	0.15	-	-	-	-	-
7. [19]	Hexane:Octane:oDCB = 3:1:1	92.05	6.74	0.028	150 °C, (N_2_ and Vacuum)	-	-	~2.5	-
Our study	Hexane:oDCB = 1:2	55.75	6.58	0.046	115 °C, 30 min, (High Vacuum)	13,445	4.21	1.29	172 (LT_50_)

**Table 2:** List of solution properties of the organic solvents. In these experiments, hexane and oDCB were used in the cosolvent process because the solution properties between the two solvents are complementary. Solvent information was referenced from PubChem (U.S. National Library of Medicine). (B.P.: boiling point, V.P.: vapor pressure).

No.	Solvent	B.P. (°C)	η	γ	ρ	V.P. (cm·Hg)
1	Hexane	69	0.003	17.89	0.66	12.4
2	Methylbenzene (Toluene)	110.6	0.0056	27.73	0.87	2.31
3	Octane	131.7	0.0073	23.93	1.11	14.05
4	Chlorobenzene (CB)	131.7	0.0073	23.93	1.11	0.88
5	1,3–Dichlorobenzene mDCB)	172	0.0104	36.2	1.29	0.138
6	1,4–Dichlorobenzene (pDCB)	174	0.00839	31.4	1.458	0.174
7	Decane	174.3	0.0085	23.37	0.73	1.43
8	1,2–Dichlorobenzene (oDCB)	180.5	0.0132	36.63	1.31	0.136
9	Ethylene Glycol	197.6	0.162	47.3	1.1132	0.006
10	Cyclohexylbenzene (CHB)	240.1	0.0269	33.29	0.95	0.004
11	a-chloronaphthalene (ClNaph)	259.3	0.0293	39.23	1.19	0.003

**Table 3:** Re number and Oh number of the cosolvent inks depending on the mix ratio of hexane and oDCB.

		Calculated Value			Measured Value		
No.	Solvent Mix rRtio	η (g/cm·s)	Re	Oh	η (g/cm·s)	Re	Oh
1	Hexane-Only Ink	0.0031	132.01	0.019	0.0038	105.88	0.024
2	Hexane:oDCB = 1:1	0.0056	72.73	0.035	0.0092	64.31	0.039
3	Hexane:oDCB = 1:2	0.0073	66.67	0.038	0.0117	55.75	0.046
4	oDCB-Only Ink	0.0132	59.27	0.043	0.0147	53.31	0.048

## Supplementary Materials

The following are available online at https://www.mdpi.com/1996-1944/13/21/4754/s1, Figure S1: Temperature-pressure phase diagram for solvent. This is an overview of how to lower the boiling point of solvent by lowering the pressure in the space around the solvent.
