# Supplementary material for: Sequential Improvement from Cosolvents Ink Formulation to Vacuum Annealing for Ink-Jet Printed Quantum-Dot Light-Emitting Diodes
Source: Materials (Basel). 2020 Oct 24;13(21):4754. doi: 10.3390/ma13214754 (PMC7660697; doi:10.3390/ma13214754)
Supplement: Supplementary file 1 [file materials-13-04754-s001.pdf]

materials

Sequential Improvement from Cosolvents Ink Formulation to Vacuum Annealing for Ink-Jet Printed Quantum-Dot Light-Emitting Diodes

Young Joon Han [1,2], Do Yeob Kim [1,2], Kunsik An [1], Kyung-Tae Kang [1], Byeong-Kwon Ju [2,*] and Kwan Hyun Cho [1,*]

[1] Manufacturing Process Platform Research and Development Department, Korea Institute of Industrial Technology (KITECH), 143 Hanggaul-ro, Sangnok-gu, Ansan-si 15588, Korea; youngjhan@kitech.re.kr (Y.J.H.); ehduq077@kitech.re.kr (D.Y.K.); kunsik1214@kitech.re.kr (K.A.); ktkang@kitech.re.kr (K.-T.K.)

[2] Department of Electrical and Electronics Engineering, College of Engineering, Korea University, 145 Anam-ro, Seongbuk-gu, Seoul 02841, Korea

* Correspondence: bkju@korea.ac.kr (B.-K.J.); khcho@kitech.re.kr (K.H.C.)

Received: 28 September 2020; Accepted: 22 October 2020; Published: 24 October 2020

1. Supplementary Materials

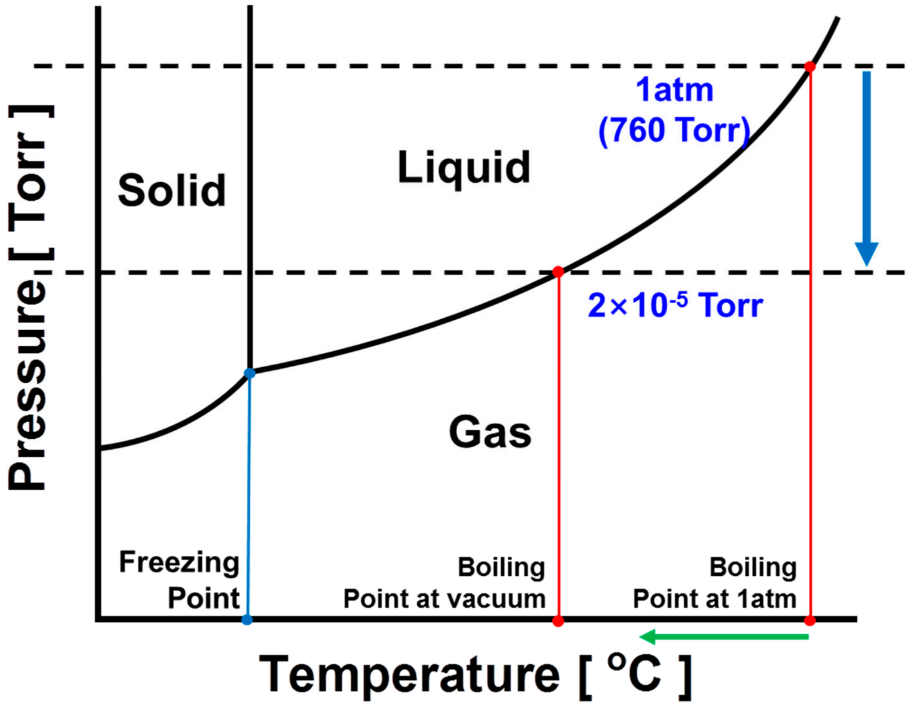

Figure S1. Temperature-pressure phase diagram for solvent. This is an overview of how to lower the boiling point of solvent by lowering the pressure in the space around the solvent.

© 2020 by the authors. Licensee MDPI, Basel, Switzerland. This article is an open access article distributed under the terms and conditions of the Creative Commons Attribution (CC BY) license (http://creativecommons.org/licenses/by/4.0/).
